# Rhein for treating diabetes mellitus: A pharmacological and mechanistic overview

**DOI:** 10.3389/fphar.2022.1106260

**Published:** 2023-01-09

**Authors:** Tingting Deng, Jinxin Du, Ying Yin, Baorui Cao, Zhiying Wang, Zhongwen Zhang, Meina Yang, Jinxiang Han

**Affiliations:** ^1^ College of Traditional Chinese Medicine, Shandong University of Traditional Chinese Medicine, Jinan, China; ^2^ Affiliated Hospital of Shandong University of Traditional Chinese Medicine, Jinan, China; ^3^ NHC Key Laboratory of Biotechnology Drugs (Shandong Academy of Medical Sciences), Biomedical Sciences College, Shandong First Medical University, Jinan, China; ^4^ Department of Endocrinology and Metabolism, The First Affiliated Hospital of Shandong First Medical University, Jinan, China

**Keywords:** rhein (PubChem CID: 10168), Rheum palmatum L. (dahuang), diabetes, blood glucose, inflammatory, oxidative stress

## Abstract

With the extension of life expectancy and changes in lifestyle, the prevalence of diabetes mellitus is increasing worldwide. *Rheum palmatum* L. a natural botanical medicine, has been used for thousands of years to prevent and treat diabetes mellitus in Eastern countries. Rhein, the main active component of rhubarb, is a 1, 8-dihydroxy anthraquinone derivative. Previous studies have extensively explored the clinical application of rhein. However, a comprehensive review of the antidiabetic effects of rhein has not been conducted. This review summarizes studies published over the past decade on the antidiabetic effects of rhein, covering the biological characteristics of *Rheum palmatum L.* and the pharmacological effects and pharmacokinetic characteristics of rhein. The review demonstrates that rhein can prevent and treat diabetes mellitus by ameliorating insulin resistance, possess anti-inflammatory and anti-oxidative stress properties, and protect islet cells, thus providing a theoretical basis for the application of rhein as an antidiabetic agent.

## Introduction

The diabetes mellitus (DM) epidemic is a global public health problem that imposes a serious social and economic burden. According to the International Diabetes Federation, DM affects an estimated 537 million adults (aged 20–79 years) worldwide, resulting in 6.7 million deaths (International Diabetes Federation, 2021). The national cost of DM in the United States (US) in 2017 was more than $327 billion, up from $245 billion in 2012 (American Diabetes Association, 2018). Hyperglycemia can cause multiple complications, such as diabetic retinopathy, diabetic nephropathy, atherosclerosis, cardiovascular disease, obesity, hypertension, hyperlipidemia, cerebrovascular disease, skin complications, depression, and ultimately death. The United States National Vital Statistics System stated that DM was the 8TH leading cause of death in the United States in 2020 (Ahmad et al., 2021). Timely control of blood sugar with effective medications is important. In addition to traditional antidiabetic drugs and insulin therapy, natural compounds isolated from herbal medicines have unique advantages and great potential in preventing and treating DM (Yang et al., 2011). The World Ethnobotanical Inspection reports approximately 800 plant species that are used to treat DM (Deniyi et al., 2018; Khursheed et al., 2019).

Rhubarb is the root and rhizome of *Rheum palmatum* L*.* It has been widely used for treating obesity (Bati et al., 2020), DM (Cheng et al., 2019), chronic kidney disease (Dou et al., 2020), pneumonia (Shen et al., 2020), osteoarthritis (Ding et al., 2018), and constipation (Gao, 2021), among others. Rhein (4,5-dihydroxyanthraquinone-2-carboxylic acid) is an anthraquinone compound extracted from rhubarb, a traditional Chinese medicine. Rhein is commonly used to treat DM in Asian and European countries (Barreto and Sahebkar, 2021). The aim of this review was to elucidate the antidiabetic effects and pharmacological mechanisms of rhein.

## Biological characteristics of Rheum palmatum L

### Nomenclature of Rheum palmatum L

**FIGURE 1:**
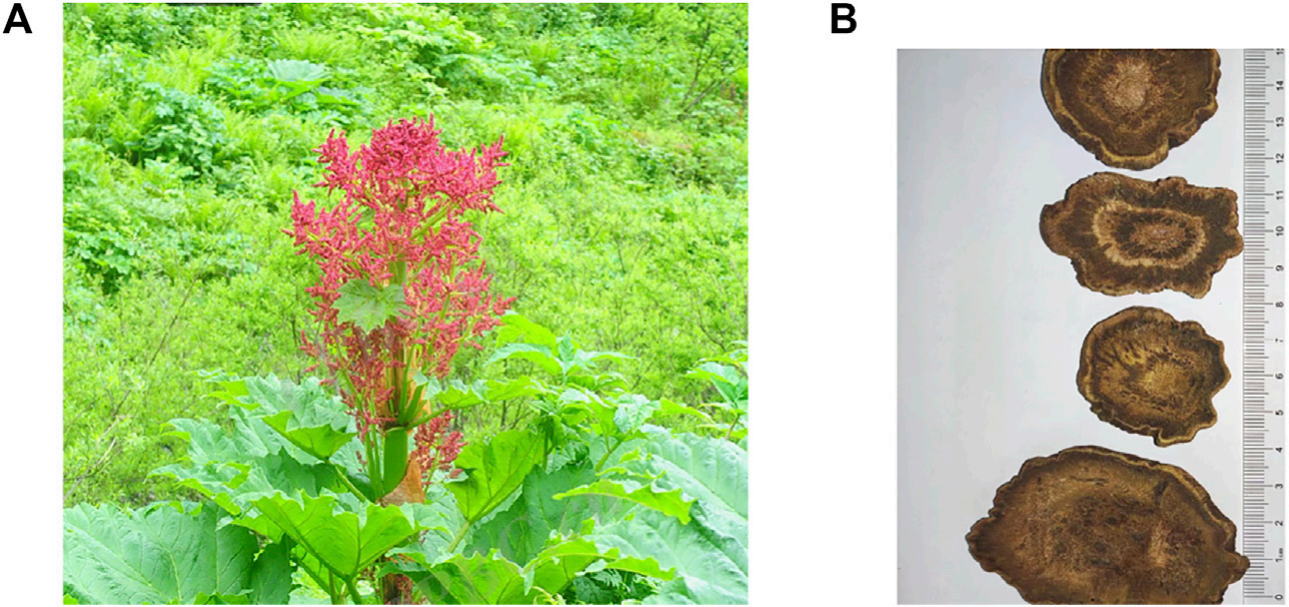
Plant and Slice of root of Rheum palmatum L. The leaves of Rheum palmatum L. are palmately lobed to half-lobed, with the lobes mostly narrowly triangular; its flowers are small, purple-red or yellowish-white; its buds are inverted pyramidal; and its fruits are small. The outer skin of rhubarb is yellowish-brown or reddish-brown, with white-like reticulate texture and scattered stars, and its broken surface is light reddish-brown or yellowish-brown, showing granularity; it has a fresh aroma and a bitter and slightly astringent taste. **(A)** Rheum palmatum L. is a kind of herb with medicinal value. **(B)** prepared slices of Rhubarb, containing a variety of active pharmacological ingredients.

*Rheum palmatum L.* is a perennial herb that belongs to the Polygonaceae family. There are approximately 60 species worldwide, mainly distributed on mountain slopes or valley wetlands at an altitude of 1,500–4,400 m. The center of distribution of this genus is China, with 41 species and four varieties, accounting for approximately 75% of the total plants in the genus. Rhubarb has been used in medicine for over 1,000 years. The traditional Chinese medicine (TCM) theory holds that rhubarb is bitter and cold and has the function of reducing accumulation, clearing heat, and eliminating fire; cooling and detoxifying blood; and removing blood stasis and meridian obstruction (Peigen et al., 1984). The detailed characteristics of *Rheum palmatum* L. and rhubarb are shown in Figure 1.

### Botanical characteristics

*Rheum palmatum L.* is a tall stout herb, 1.5–2.0 m high, with thick woody roots and rhizomes. The stem is upright and hollow, and the leaves are nearly equal in length and width, up to 40–60 cm long. The apex is narrow and pointed, and the base is nearly heart-shaped, usually palmately hemi-5-lobed. The petiole is stout, cylindrical, nearly equal in length to the leaf, and densely covered with rusty papillary hairs. Cauline leaves get smaller upwards, while the stalks get shorter. The leaf sheath is large, up to 15 cm long, with a smooth inner surface and a rough exterior. The inflorescences are large, conical, branched, and more clustered, densely covered with coarse short hairs. The flowers are small, usually purple-red and sometimes yellowish white. The peduncle is 2.0–2.5 mm long, and the joints are located below the middle. The fruit is rounded, oval to rectangular, 8.0–9.0 mm long, and 7.0–7.5 mm wide. The seeds are broadly ovate and brownish black. The flowering period is June, and the fruiting period is August. Rhubarb is mainly distributed in the temperate and subtropical regions of Asia.

### Rhein: The main therapeutic ingredient of rhubarb

**FIGURE 2:**
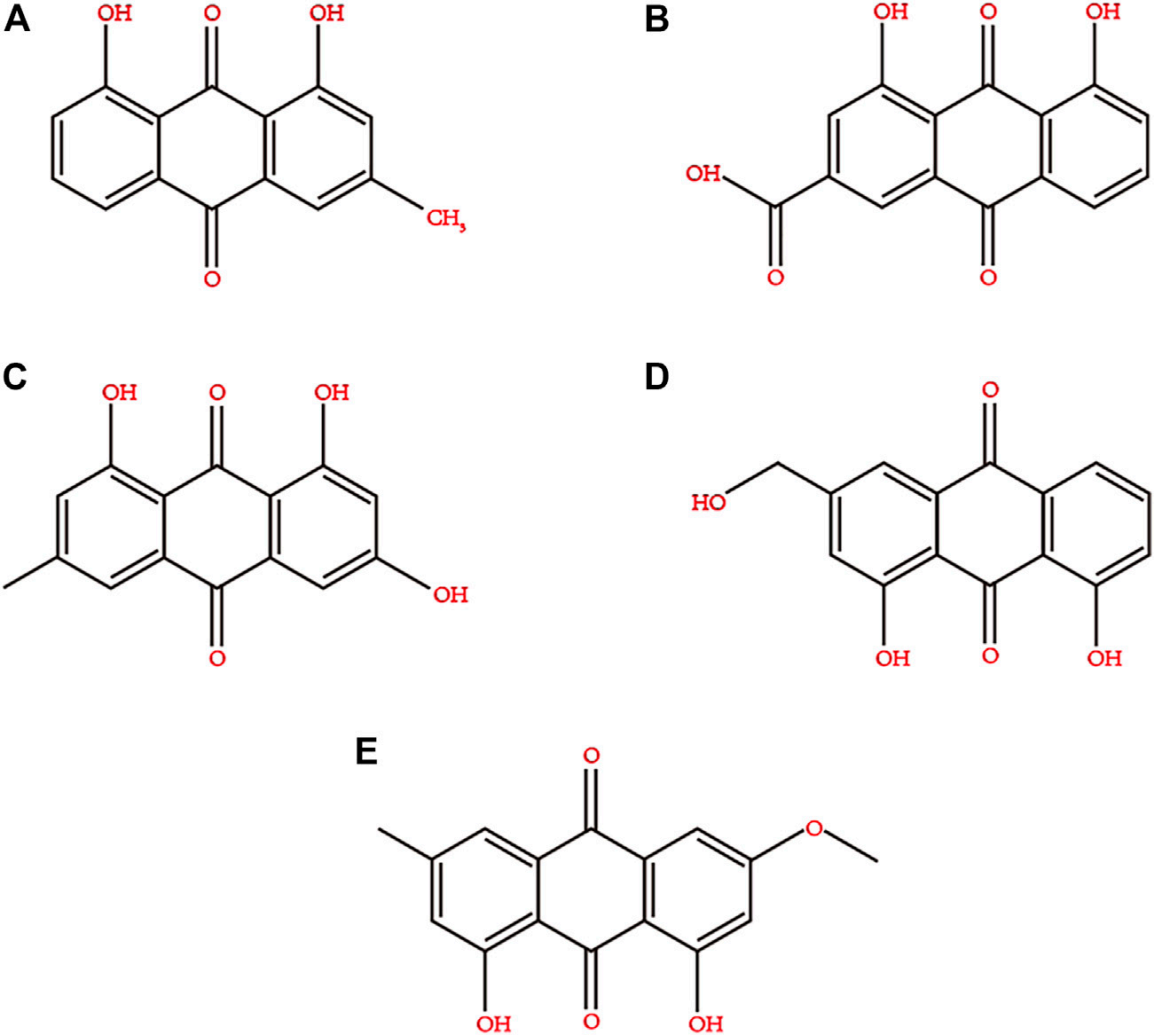
Chemical structures of the main active ingredients of Rhubarb. **(A)** chrysophanol, **(B)** rhein, **(C)** emodin, **(D)** aloe-emodin, **(E)** emodin methyl ether.

Rhubarb contains a variety of biologically active ingredients, such as anthraquinone derivatives, anthracene derivatives, styrene, tannins, acyl glycosides, chromones, and phenylbutazone glycosides (Pharmacopoeia of the People’s Republic of China 2005). However, the main ones are rhein, emodin, aloe-emodin, chrysophanol, and emodin methyl ether (Ye et al., 2007). The chemical structures of these compounds are depicted in Figure 2. The extraction methods for the active ingredients include marinated extraction, heat reflux extraction (HRE), Soxhlet extraction (SE), and microwave-assisted extraction (MAE) (Sun and Liu, 2008; Huang et al., 2009). In the 21st century, rhubarb has been used in various medicinal and edible applications (Rhubarb, 2006). There is increasing evidence that rhein is effective against DM and its complications and can act on different anti-diabetic drug targets, indicating its potential as an anti-diabetic agent (Qi et al., 2010; Zeng et al., 2014; Chien et al., 2015; Zheng, 2020).

### Extraction methods and quality control

The extraction methods of rhein include ultrasound-assisted extraction (UAE), HRE SE, and MAE (Sun and Liu, 2008; Huang et al., 2009). The UAE method has the advantages of high extraction efficiency, short consumption time, and low temperature. The HRE method is suitable for small amounts of drugs that aren’t easily destroyed by heat. The SE method is easy to operate, and the MAE method has the characteristics of uniform and selective heating.

In recent years, proton ionic liquids have been used as efficient solvents for MAE of rhein (Fan et al., 2019). A variety of qualitative and quantitative analytical methods have been developed for determining rhein content, including thin layer chromatography (Ma et al., 1989), high performance liquid chromatography (HPLC)-ultraviolet detection (Zhou et al., 2009), HPLC-mass spectrometry (Danielsen and Francis, 1994), high performance capillary electrophoresis (Hu, 2012), and capillary electrophoresis chromatography (Koyama et al., 2007). According to the provisions of the 2020 edition of the Chinese Pharmacopoeia (Chinese Pharmacopoeia Commission, 2020), the total ash content of medicinal materials should not exceed 10.0% (General Rule 2302). The total anthraquinone content was determined using HPLC (General Rule 0512), with the total amount of aloe emodin (C_15_H_10_O_5_), rhein (C_15_H_8_O_6_), emodin (C_15_H_10_O_5_), chrysophanol (C_15_H_10_O_4_), and emodin methyl ether (C_16_H_12_O_5_) not less than 1.5%. It shouldn’t be less than 25.0% according to the hot-dip method, which is a water-soluble extract determination method (General Rule 2201).

## Pharmacological mechanisms of rhein on DM mellitus

### Insulin resistance

Insulin resistance (IR) is a common risk factor for various endocrine, metabolic, and cardiovascular diseases (Zhang et al., 2019; Hill et al., 2021). Rhein can significantly improve IR associated with obesity (Ji and Gu, 2021) and non-alcoholic fatty liver disease (Sheng et al., 2011). Similarly, rhein can play a role in reducing IR, thereby inhibiting DM progression.

Insulin resistance prevents insulin from performing its normal physiological function, causing high blood glucose. This results in hyperinsulinism and a series of changes such as hyperglycemia and obesity. Rhein significantly improves glucose tolerance without regulating insulin secretion in streptozotocin (STZ)-induced diabetic mice (Choi et al., 2006). It was found to significantly reduce blood glucose, glycosylated hemoglobin, and glycosylated serum protein levels, increase the insulin sensitivity index, and decrease the IR index, Homeostatic Model Assessment for Insulin Resistance, effectively improving insulin sensitivity in diabetic rats (Jin, 2008). These findings confirm that rhein enhances insulin sensitivity.

The hexosamine pathway (HBP) is an intracellular glucose metabolism pathway. Under normal circumstances, only 1%–3% of glucose enters the HBP. Changes in intracellular HBP activity are associated with the development of IR (Marshall et al., 1991; Cooksey et al., 1999). Studies have shown that rhein inhibits the development of DM mainly by inhibiting the overactivity of HBP. Specifically, after rhein stimulation, the activity of glutamine fructose-6-phosphate aminotransferase (GFAT) in the first step of the HBP decreases, and the formation of UDP-N- acetylglucosamine, the final product of HBP, decreases significantly (Liu et al., 2000; Liu et al., 2001a). Glucose transporter 1 (GLUT1) is the key glucose transporter in mesangial cells and is responsible for their basal metabolism; its expression level is often the main rate-limiting step in cellular glucose metabolism. Modern research has found that rhein can significantly reduce the glucose uptake rate of mesangial cells were transduced with the human GLUT1 gene (MCGT1) in a dose-dependent manner, correct the hypertrophic state of MCGT1, and reduce the cell volume of MCGT1 and the ratio of RNA/DNA and protein/DNA (Liu et al., 2001a; Li et al., 2001; Zhu et al., 2001).

**FIGURE 3:**
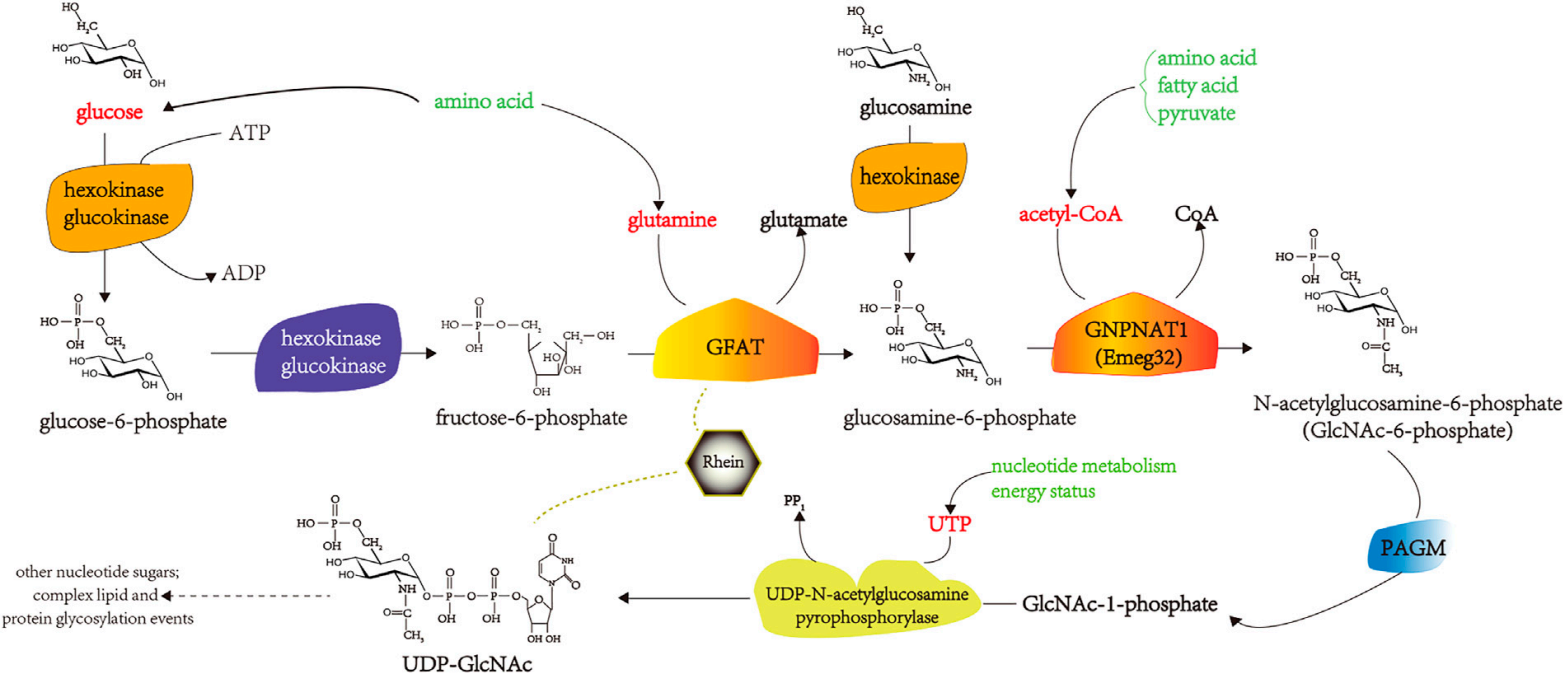
The pharmacological mechanisms of rhein in improving IR in DM by inhibiting HBP overactivity. PAGM, Phosphoacetllucosaminemutase; ATP, adenosine triphosphate; ADP, adenosine diphosphate; UDP, uridine diphosphate; UTP, uridine triphosphate.

In mesangial cells, transforming growth factor-β (TGF-β1) stimulates extracellular matrix (ECM) synthesis and cell hypertrophy as a result of high glucose concentrations (Masson et al., 2005; Masson et al., 2006). Rhein inhibits the overactivity of HBP in rat mesangial cells (MCGT1) transfected with the GLUT1 gene. It reduces the expression of TGF-β1, number of hypertrophic cells, and ECM synthesis (Liu et al., 2001b; Zheng et al., 2008). Rhein inhibits the upregulation of plasminogen activator inhibitor-1 (PAI-1) mRNA expression in TGF-β1-induced endothelial cells in a dose-dependent manner and the production of endothelial PAI-1 protein and has a significant inhibitory effect on the activity of phosphor-p44/p42 mitogen-activated protein kinase (MAPK) induced by TGF-β1 in human endothelial cells (Zhu et al., 2003). Rhein is transmitted through protein kinase C (PKC), and MAPK inhibits the abnormal increase in GLUT1 mRNA expression, thereby antagonizing the pathological effects of TGF-β1 on mesangial cells (Huang et al., 2007; Zhuang et al., 2019). GFAT is the key enzyme in HBP, and its activity intensity reflects the active state of HBP. Liu Q. found that rhein inhibits the activity and expression of GFAT in the skeletal muscle of IR mice, 3T3 L1 adipocytes, and C2C12 myoblast models of IR induced by high glucose and insulin levels, thereby inhibiting HBP flow and downregulating the glycosylation modification level of the protein O-linked N-acetylglucosamine. It increases the responsiveness of the whole body and insulin target tissues to insulin (Liu Q., 2009). The pharmacological mechanisms by which rhein improves IR in DM by inhibiting HBP overactivity are shown in Figure 3.

In addition, rhein can increase the expression of *SIRT1* in the renal tissue, improve insulin sensitivity and glucose and lipid metabolism disorders, and reduce the IR index in diabetic rats (Chen et al., 2015). A study found that argirein synthesized by hydrogen bonding of rhein and L-arginine inhibits the activation of NOX4-dependent O_2_^−^ in rat aortic endothelial cells triggered by palmitic acid, thereby inhibiting endothelial IR and improving vascular function (Li et al., 2018). Rhein significantly reduces blood glucose levels in diabetic mice and upregulates peroxisome proliferator-activated receptor *γ* (PPARγ) expression, thus improving metabolic disorders and reversing IR (Huang et al., 2004; Chi et al., 2008).

### Inflammation

The risk of developing DM is positively associated with inflammation (Navarro and Mora, 2005; Shoelson et al., 2006). Rhein exhibits significant anti-inflammatory properties in colitis (Dong et al., 2022; Zhou et al., 2022), acute pancreatitis (Yang D et al., 2022), and obesity (Fang et al., 2022), among other conditions. Similarly, rhein exerts anti-inflammatory effects by inhibiting DM development. Inflammation induces the release of chemicals called inflammatory cytokines from damaged cells; these cytokines regulate various inflammatory responses. Studies have shown that rhein inhibits the development of DM primarily by inhibiting the release of inflammatory cytokines. In the KK/HlJ diabetic mouse model, rhein lysinate reduces the levels of blood glucose and significantly decreases the expression of tumor necrosis factor alpha (TNF-α) and interleukin 6 (IL-6) (Wei et al., 2016). Another study reported that Rhein@OR-S gel can inhibit reactive oxygen species (ROS) and NO overproduction and has excellent efficiency in suppressing diabetic wound inflammation, bringing forward the transition from the inflammatory phase to the proliferative phase (Zhao et al., 2020).

Nuclear factor-kappa B (NF-κB) is a sequence-specific DNA-binding protein with pleiotropic transcriptional regulation, regulating the expression of multiple target genes encoding cytokines and inflammatory mediators. The NF-κB signaling pathway plays an important role in the molecular mechanisms of IR. After stimulation by exogenous substances, such as TNF-α, IL-1β, and LPS, IκB-α in the cytoplasm is phosphorylated, undergoes ubiquitination and degradation, and is released into p65 into the nucleus. In turn, p65 binds to specific response element sequences on target genes and activates their transcription (Pires et al., 2018). Rhein plays an anti-inflammatory role by inhibiting the NF-κB signaling pathway through the degradation of IκB-α and nuclear translocation of p65 (Li and Verma, 2002; Tobar et al., 2011; Zheng et al., 2019; He et al., 2021). Macrophages exacerbate inflammatory responses by releasing inflammatory factors. Rhein plays a dual role in lipopolysaccharide-activated macrophages by inhibiting IKKβ. On the one hand, by inhibiting the standard IKKβ-mediated NF-κB activation sequence (IKKβ axis), rhein suppresses downstream NO and IL-6 levels; on the other hand, rhein enhances caspase-1 activity by inhibiting NF-κB-independent IKKβ itself, which in turn increases the release of IL-1β and HMGB1 (Gao et al., 2014). PPARγ is a significant protein involved in insulin activity. PPAR-γ ligand downregulates the expression of TNF-α in macrophages PPAR-γ (Bermudez et al., 2017). C-Jun N-terminal kinase (JNK) is a member of the mitogen-activated protein kinase (MAPK) family, which is a critical component of the death pathway caused by inflammation and oxidative stress. Rhein can regulate the PPARγ signaling pathway in a dose-dependent manner, effectively improving the protein expression of PPARγ, and inhibiting NF-κB expression and p-JNK activation. These changes effectively inhibit the inflammatory response of renal mesangial cells and enhance insulin sensitivity (Duan S. F. et al., 2016; Ji and Gu, 2021).

The Wnt/β-catenin signaling pathway activated by high glucose levels is involved in the pathogenesis of DM (Park et al., 2011; García-Jiménez et al., 2013). *ß*-Catenin is mainly responsible for mediating intercellular adhesion and related signaling. Stimulation of the Wnt signaling pathway is the only mechanism that enhances *ß*-catenin stability (Lin, 2004). When the Wnt signaling pathway is activated, the Wnt protein binds to the receptor, thereby inhibiting the activity of GSK-3β. Thus, *ß*-catenin cannot be phosphorylated and degraded, then accumulates in the cytoplasm, and integrates into the nucleus to initiate the expression of downstream target genes. Rhein can downregulate the expression of *ß*-catenin protein, promote the expression of GSK-3β, reduce the secretion of TGF-β1, and play a role in inhibit the Wnt/β-catenin signaling pathway (Zhang et al., 2015; Duan S. et al., 2016). The NLRP3 inflammasome is involved in inflammation and glucose homeostasis, and its activation can induce immune cells to produce proinflammatory cytokines, thereby exacerbating IR. NLRP3 inflammasome provides a platform for the production of IL-1β. IL-1β directly inhibits the insulin signaling pathway by decreasing insulin receptor substrate-1 (IRS-1) tyrosine phosphorylation and negatively regulating IRS-1 gene expression (Ding et al., 2019). Rhein inhibits NLRP3 inflammasome activation (Ge et al., 2017).

Rhein analogs may also improve diabetic symptoms by reducing the production of pro-inflammatory cytokines and inhibiting the expression of TGF-β1. Rhein lysinate (RHL), a water-soluble rhein derivative, can reduce the expression of TNF-α and NF-κB, and inhibits the immune response by directly or indirectly blocking the TNF–NF-κB biochemical pathway (Lin et al., 2015; Lin et al., 2017; Chueakula et al., 2018). Diacerhein is completely metabolized in the human body, resulting in rhein production, which exerts anti-inflammatory properties locally, decreases the expression of pro-inflammatory cytokines such as IL-1β, TNF-α, IFN-γ, and IL-12, thereby interfering with the occurrence of DM (Malaguti et al., 2008). Diacerein can reduce the level of TNF-α in patients with DM and improve metabolic control (Piovesan et al., 2017; Tres et al., 2018). The experimental details of the anti-inflammatory mechanisms of rhein are presented in Table 1.

**TABLE 1:** Experiments on the anti-inflammatory mechanism of rhein in diabetes mellitus.

Authors	Experimental model	Experimental method	Signaling molecules involved (rhein group)
Ji et al.	Obesity model of immature female rats induced by high fructose diet	HE staining, Tunel assay, Western blot, Cell culture, Oil red staining, Flow cytometry, Immunofluorescence assay	IL-6↓, IL-1β↓, TNF-α↓, ROS↓, MDA↓, SOD↑
Duan et al.	High glucose induced MsC	MTS, ELISA, RT-PCR	TGF-β1↓, MCP-1↓
Lin et al.	high-fat diet and STZ induced diabetic mice	Western blot, Nile red staining	TNF-a↓, IL-6↓, ERK1/2↓, SREBP-1c↓, SOD↑, GSH-Px↑
Malaguti et al.	NOD mice	Total RNA extraction, RT-PCR, ELISA, HPLC	IL-1β↓, IL12↓, IFN-γ↓, TNF-α↓
Lin et al.	KK/HlJ mice	urinary albumin, Measurement of laboratory parameters in serum, Western blot, antioxidant activity, Histological and immunohistochemical analysis	ACR↓, blood glucose↓, creatinine↓, urea↓, SOD↑, GSH-px↑, MDA↓
Wei et al.	RHL-treated KK/HlJ mice	Hepatic lipid analysis, Western blot	TNF-a↓, IL-6↓, NF-κB↓, MDA↓, SOD↑, GSH-px↑
Zhang et al.	human mesangial cells	MTT measurement, ELISA Immunohistochemistry	TGF-β1↓, Wnt↓, *ß*-catenin↓
Duan et al.	the rats’mesangial cell (MsC) induced by high glucose	Cell survival rate, RT-PCR, Western blot	p-JNK↓, Bcl-2↓, PPARγ↑
Tobar et al.	DIO mice	Protein analysis by immunoblotting,Real-time PCR, Morphometry	TNF--α↓, IL-6↓, IL-1β↓, IKKβ↓, JNK↓, PTP1B↓, PTP1B↑
Chueakula et al.	high-fat diet mice	Determinations of renal function, Determination of renal Oat3 function and expression, Determination of MDA level in renal cortex, Histopathological studies, OGTT, Tail-cuff BP measurement, Blood parameter analysis, western blot	NF-κB↓, IL-6↓, IFN-γ↓,TNF-αR1↓, Nrf2↓, Oat3↑, MDA↓, PKCα↓, HO-1↓, AT1R↓, SOD2↑
Piovesan et al.	Type 2 DM participants with CKD	HOMA-IR, indexenzymatic and colorimetric method, Jaffe method, ELISA, immunoturbidimetric assay, immunofluorescence assay	TNF-α↓
He et al.	type 2 diabetic rats induced by a high-fat diet and streptozotocin	Insulin tolerance testing, qRT-PCR, Western blot, Vascular reactivity experiments	IL-1β↓, TNF-a↓, IL-6↓, p-eNOS↑,iNOS↓,p-p65↓, p-IkBa↓, NLRP3↓, ASC↓, Caspase-1↓, AUC-ITT↓
Tres et al.	Patients with T2DM	Luminex^®^ Human Ultrasensitive magnetic bead panel, enzymatic and colorimetric method, immunoturbidimetric assay	HbA1c↓, TNF-α↓, IL-1β↓

### Oxidative stress

Under high glucose conditions, autoxidation of glucose, non-enzymatic glycosylation of glucose and other biomolecules, and enhancement of polyol metabolic pathways results in the production of large amounts of ROS in the body. The inhibition of antioxidants (vitamin C, vitamin E, etc.*,*) and antioxidant enzymes (SOD, glutathione (GSH, catalase, etc.*,*) used to scavenge ROS in the body leads to the accumulation of lipid peroxidation products (e.g., MDA) and a long-term imbalance between the production of highly reactive molecules and antioxidant effects, resulting in oxidative stress, which in turn activates the polyol, AGEs, and PKC pathways, causing IR and impairment of islet *ß*-cell function (Scott and King, 2004; Pitocco et al., 2010).

**FIGURE 4:**
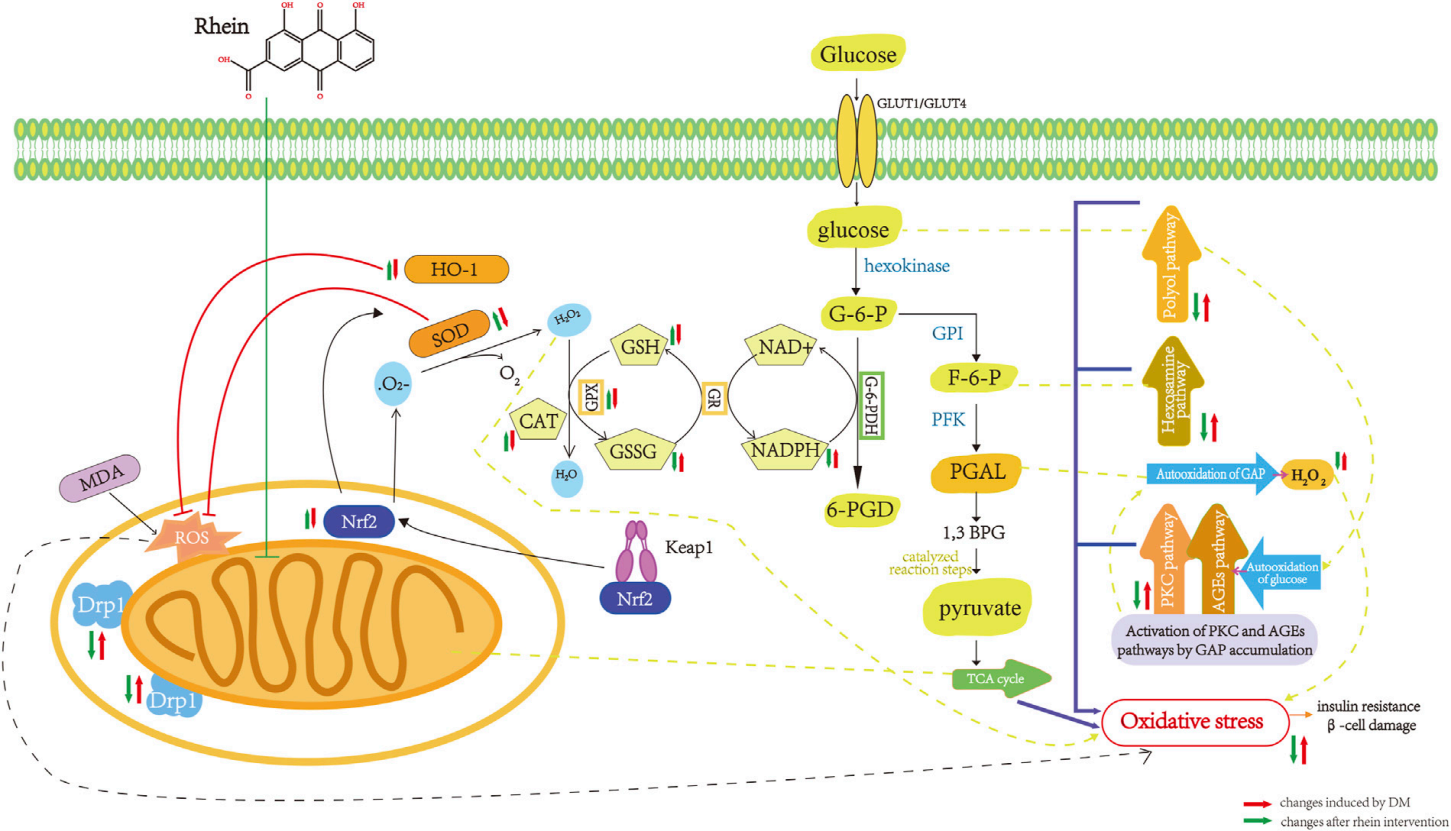
Pharmacological mechanism of rhein in inhibition of oxidative stress in diabetes mellitus. Drpl, dynamin-relatedprotein 1; Nrf2, nuclea factor erythroid-2-related factor 2; MDA,malonaldehyde; ROS, reactive oxygen species; SOD,superoxide dismutase; HO-1, Heme Oxygenase-1; CAT,catalase; GPX, glutathione peroxidase; GSH, glutathione,reduced; GSSG glutathione,oxydized; GR, gluathione reductase; NADPH, nicotinamide adenine dinucleotide phosphate; G-6-PDH,glucose-6-phosphate dehydrogenase; G-6-P, glucose-6-phosphate; GPI, phosphohexose isomerase; F-6-P, fructose-6-phosphate; PEK, Phosphofructokinase; PGAL, glyceraldehyde-3-phosphate; 1,3 BPG, 1,3 Biphosphoglycerate; TCA cycle, tricarboxylicacidcycle.

Rhein has a unique structure containing carboxyl and hydroxyl groups, including two hydroxyl radicals, which confer antioxidant properties. Rhein inhibits OS in histiocytes by inhibiting antioxidant enzymes and reducing coenzyme II (NADPH) oxidase, reducing peroxisomes such as ROS *in vivo*, and has a protective effect on islet function and renal histiocytes. Huang et al. applied rhein to treat STZ-induced DM rats and found that rhein could antagonize the effect of Ang II and inhibit the signaling pathway of Ang II receptor, significantly inhibit the mRNA expression of renal NADPH oxidase subunits p47^phox^ and p22^phox^. This reduces the production of ROS, which increases the antioxidant capacity of kidney tissue in DM and inhibits the oxidative emergency of kidney tissue (Huang et al., 2012). Huang et al. reported that rhein could reduce the expression of 8-OHdG, a marker of DNA damage, significantly inhibit OS injury in islet cells, and protect islet function (Huang et al., 2013). Rhein inhibited the increase in MDA content, increased SOD activity, and significantly inhibited OS damage in pancreatic islet cells (Wang J B et al., 2011; Li and Zhen, 2017). The pharmacological mechanism of rhein in inhibition of oxidative stress in DM are shown in Figure 4.

### Gut microbiota

Gut microbiota dysbiosis leads to an increase in the proportion of G-bacteria, which, through the production and uptake of more LPS, activates a low-grade chronic inflammatory response, exacerbates IR, and impairs *ß*-cell function (Larsen et al., 2010). A two-stage metagenome-wide association study based on deep next-generation shotgun sequencing of gut microbial DNA extracted from 345 individuals identified alterations in vitamin and cofactor synthesis-related, butyrate-producing, sulfate-reducing, and mucin-degrading bacteria in the gut of patients with DM. The gut environment of patients with DM is one that stimulates bacterial defense mechanisms against OS (Qin et al., 2012). The ratio of *Bacteroidetes* to *Firmicutes* was positively correlated with blood glucose concentration. Rhein significantly enriched the gut microbiota, increased the relative abundance of *Bacteroides*, reversed the ratio of *Bacteroides* and *Firmicutes*, maintained the diversity of the gut microbiota to improve glucose metabolism, and exerted a hypoglycemic effect (Wang et al., 2016; 2018). Rhein significantly increased the number of terminal ileal L cells in db/db mice, which increased glucagon-like tide-l (GLP-1) release, which in turn stimulated increased insulin secretion by islet *ß*-cells and inhibited glucagon secretion by α-cells, thus improving glucose homeostasis (Wang et al., 2016).

### Dyslipidemia

Diabetic dyslipidemia, which comprises very-low-density lipoprotein (VLDL), triacylglycerol (TG), small dense low-density lipoprotein, low-density lipoprotein (LDL), and high-density lipoprotein (HDL), is common in patients with DM (Bahiru et al., 2021). Multiple polymorphic studies have confirmed that hyperglycemia and elevated plasma triglycerides are associated with hepatic steatosis (Liang et al., 2015; Zhu et al., 2020). 3-hydroxy-3-methyl glutaryl coenzyme A (HMG-CoA), a rate-limiting enzyme in cholesterol synthesis by hepatocytes, catalyzes the formation of mevalonate. Gao et al. found that rhein inhibits HMG-CoA reductase, thereby inhibiting cholesterol synthesis (Gao et al., 2010). Wu et al. found that rhein significantly reduced the contents of TG and TC in L02 hepatic steatosis cells, and significantly reduced the fat granules around the nucleus (Wu et al., 2022). Rhein can downregulate the expression of resistin in the adipose tissue of obese diabetic rats, thereby reducing plasma free fatty acid levels and inhibiting signal transduction to insulin (Liu et al., 2011). Under pathological conditions such as DM and fatty liver, oxidized low-density lipoprotein can induce an increase in PPAR-γ through the PKC-α/ERK/PPAR-γ pathway (Hashimoto et al., 2000; Neuschwander-Tetri and Caldwell, 2003), which can inhibit the susceptibility of PPAR-γ and the expression of its target genes in a dose-dependent manner (Cen et al., 2013). Rhein can reduce serum total cholesterol (TC) levels, increase HDL levels, reduce LDL and VLDL levels, and prevent excessive oxidation of LDL in mice (Xie and Shang, 2014).

### Mitochondrial dysfunction

When the inner and outer mitochondrial membrane proton gradients are high and oxygen consumption is low, ROS generation in the body increases. Excessive ROS damage mitochondrial proteins, mtDNA, and lipids on the mitochondrial membrane, thereby causing mitochondrial damage. Sustained and high-yield ROS generation caused by mitochondrial function damage reduces ATP generation, damages *ß* cells and inhibits insulin release (Hodgin et al., 2013). ROS prevents IRS-1 from binding to the insulin receptor by activating IKKβ, which phosphorylates the IRS-1 SER site, thereby preventing the transmission of insulin signals and causing insulin resistance (Nishikawa, 2007). Rhein is localized in the mitochondria of pancreatic *ß*-cells, and it can maintain mitochondrial ultrastructure by reducing the ROS content in mitochondria, thereby inhibiting the expression of dynamin related protein 1 (Drp1) and preventing *ß*-cell apoptosis induced by hyperglycemia (Lin et al., 2007; Liu et al., 2013). AMP-activated protein kinase (AMPK) is an important protein that regulates mitochondrial biosynthesis (Jager et al., 2007). Studies have shown that rhein can activate the AMPK-Sirt1 pathway to promote the translocation of the α-subunit of ATPase to the plasma membrane, promote mitochondrial biosynthesis, improve mitochondrial respiratory function, and ultimately improve insulin resistance (Tu et al., 2017; Rhein et al., 2021).

### β-cell failure

**FIGURE 5:**
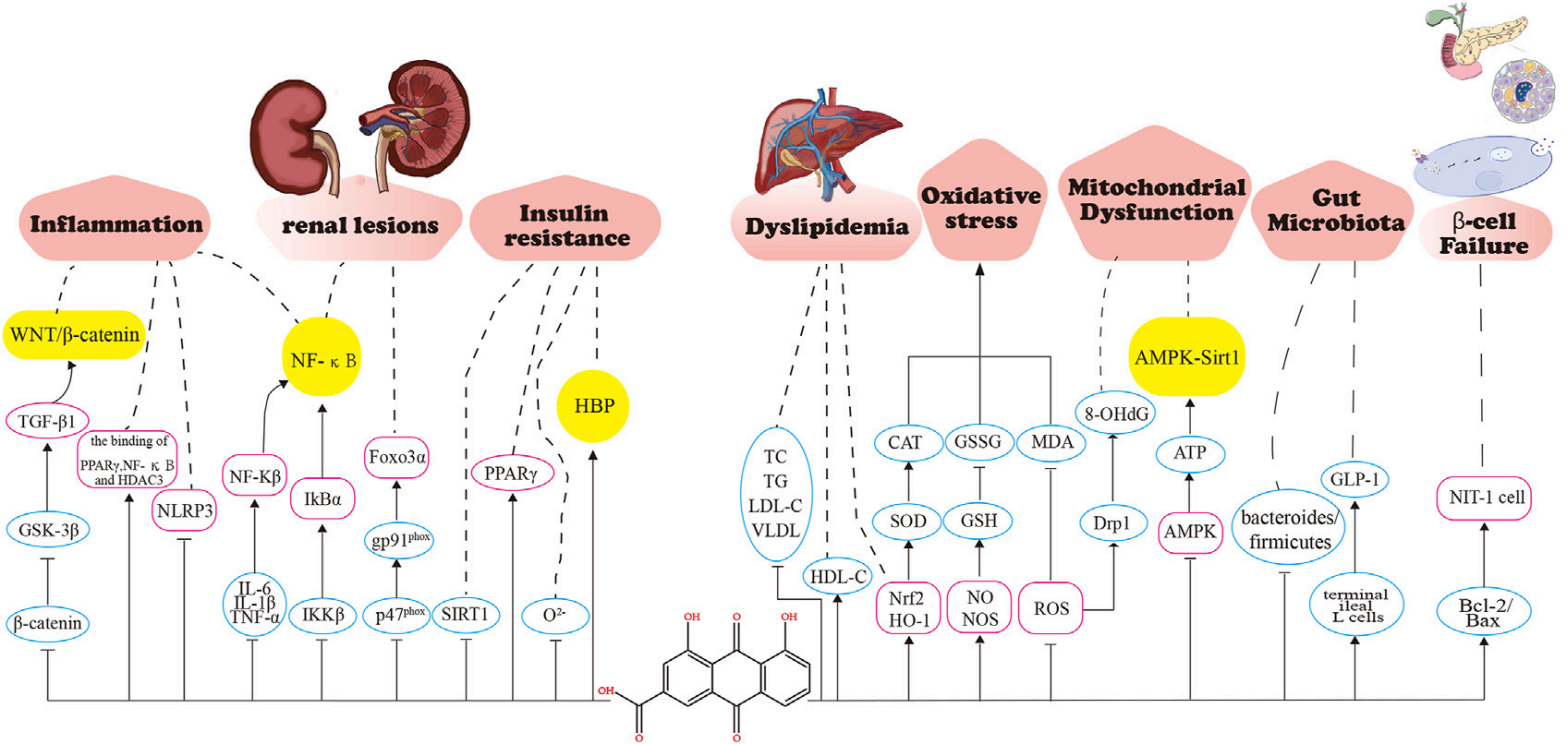
Pharmacological mechanisms of rhein in diabetes mellitus. Rhein maintains glucose homeostasis by anti-inflammation, alleviating renal lesions, improving insulin resistance, improving lipid metabolism, anti-oxidative stress, maintaining mitochondrial respiratory function, improving gut microbiota, and improving β -cell functional. GSK-3 β, Glycogen synthase kinase 3 β; PPAR- γ ,peroxisome proliferator-activated receptor γ ; NF-κ B, nuclear factor kappa-B; HDAC3, histone deacetylase 3; NLRP3, NOD-like receptor thermal protein domain associated protein 3; IL-6, Interleukin-6; IL-1 β ,Interleukin-1 β ; TNF- α ,tumor necrosis factor α ; IKK β ,inhibitor of kappa B kinase β ;Foxo3 α, Forkhead box 03 α ; SIRT1, sirtuin 1; HBP, Hexosamine pathway; TC, Serum total cholesterol; HDL-C,High density liptein cholesterol; TG, triacylglycerol; LDL-C, Low-Density Lipoprotein Cholesterol; VLDL,very low-density lipoprotein; SOD, Superoxide dismutase; CAT,Catalase from micrococcus lysodeiktic; GSH,glutathione,reduced; GSSG, glutathione, oxydized; MDA, malonaldehyde; ROS, reactive oxygen species; Drpl,Dynamin-related protein 1; AMPK,Adenosine 5' -monophosphate (AMP)-activated protein kinase; GLP-1,glucagon-like peptide-1; Bcl-2,B-cell lymphoma-2; BAX,BCL2-Associated X.

Insufficient relative or absolute insulin secretion due to an insufficient number of pancreatic *ß*-cells causes hyperglycemia (Wajchenberg, 2007). Type 2 DM is the result of progressive loss of pancreatic *ß*-cell number and secretion function (Park et al., 2012). The main mechanism for the decrease in *ß*-cell numbers is an increase in *ß*-cell apoptosis rather than a decrease in proliferation or replication (Lupi and Del Prato, 2008). Hu et al. applied H_2_O_2_ treatment to NIT-1 cells as a model of pancreatic cell damage and found that RHL increased the expression level of anti-apoptotic protein Bcl-2, decreased the expression level of pro-apoptotic protein Bax, and increased the Bcl-2/Bax ratio, which partially blocked NIT-1 cell apoptosis induced by H_2_O_2_ and protected pancreatic *ß* cells, thus exerting a pancreatic protective effect (Hu et al., 2014). Rhein significantly inhibited the reduction of *ß*-cell mass and reduced *ß*-cell apoptosis, which improved the glucose-dependent and glucose-independent secretion of insulin by improving *ß*-cell function (Du et al., 2012; Liu et al., 2013). The pharmacological mechanisms of rhein in DM are presented in Figure 5.

## Toxicology of rhein

In recent years, increasing adverse reactions due to high doses or long-term administration of rhubarb and its preparations have caused global concern (Wang M et al., 2011; Zeng et al., 2013; Cao et al., 2019). Therefore, it is crucial to determine its toxic components. Studies have shown that rhein exhibits hepatotoxicity and nephrotoxicity (Hu et al., 2019; Yang X et al., 2022). Rhein can inhibit HepaRG cell viability and induce cell death by blocking the S-phase cell cycle and activating the Fas- and mitochondria-mediated apoptotic pathways (You et al., 2018). Rhein affects the accumulation of Rh123 in hepatocyte mitochondria, thereby altering the mitochondrial membrane potential and affecting mitochondrial membrane permeability; mitochondrial dysfunction causes changes in Ca^2+^ homeostasis and ATP depletion, ultimately leading to cell death (Bironaite and Ollinger, 1997). He et al. found that CYP2C19 could activate rhein to reactive metabolites (RMs), and RMs can covalently bind to intracellular mitochondria, resulting in ROS overproduction, respiratory chain dysfunction, liver function impairment (increased aspartate aminotransferase and lactate dehydrogenase), and cell death. In addition, RM overproduction depletes GSH, leading to hepatotoxicity (He et al., 2015). Mao et al. demonstrated that rhein can induce apoptosis in HK-2 cells by activating caspase-3 and caspase-9, which are involved in the ROS-dependent mitochondrial pathway (Sun et al., 2015; Mao et al., 2017). Rhein induces apoptosis by activating caspase-3 expression, resulting in pathological changes in renal histology, mainly manifested as protein casts in the lumen of renal tubules, capillary congestion in glomeruli and renal interstitium, swelling of renal tubule epithelial cells, and small focal proliferation of lymphocytes (Hu et al., 2019). Repeated administration of rhein for 75 days resulted in obvious steatosis and cell swelling in the liver, as well as varying degrees of inflammation, swelling, and necrosis in the kidney and colon cells, associated with competitive binding of rhein to multidrug resistance-associated protein 2 (MRP2) protein sites. Reduced expression of MRP2 protein blocked cellular oxidative stress substance expulsion, causing increased oxidative stress, impaired mitochondrial function, and stimulation of the release of caspase 3, a downstream factor of apoptosis, thus exhibiting cytotoxicity (Yang, 2022).

## Pharmacokinetics of rhein

The HPLC-fluorescence detector method has been used to study the absorption of rhein in rats after oral administration, and the results showed that rhein is mainly distributed in the stomach, kidney, liver, and intestine, had good biocompatibility with them, and could pass the blood-brain barrier (Zhao et al., 2021). Rhein is mainly metabolized by the kidneys. The cumulative excretion rate in urine is 6.0 ± .6% within 24 h after oral administration of 70 mg/kg rhein, and the renal clearance of rhein is 21.3 ± 5.47 mL/h/kg (Hao et al., 2014). In total, 20% of rhein is excreted in its natural form in urine, 60% as thiourea acid ester conjugates, and 20% as sulfates (Dahms et al., 1997). Rhein is the only anthraquinone component of rhubarb that is absorbed into the blood in the human body (Lee et al., 2003).

Takizawa et al., 2003 used HPLC-ultraviolet detection to determine the plasma levels of rhein, which rapidly reached the maximum level after oral administration. In addition, the plasma concentration-time curve revealed secondary peaks, indicating enterohepatic recirculation of rhein. Zhang et al. (2013) used liquid chromatograph mass spectrometer to compare the pharmacokinetics of rhein in rat plasma after oral administration of rhubarb peony decoction (RPD) and rhubarb extract and found that the C_max_ of rhein in RPD was significantly lower than that after administration of rhubarb, indicating that the absorption of rhein in rats was inhibited after oral administration of RPD. Hou et al. (2014) applied Ultra-high performance liquid chromatography-tandem mass spectrometry to simultaneously quantify the active ingredients in commercial ready-to-use pharmaceutical herbal products to investigate the pharmacokinetics of rhein, rhubarb, and San-Huang-Xie-Xin-Tang (SHXXT) in rats and found that C_max_ and area under the curve (AUC) in the SHXXT group, compared with the rhein administration group, were significantly increased by 17-fold and 9-fold, respectively, indicating that the absorption rate of rhein *via* SHXXT was significantly higher than that of pure rhein. Based on the pharmacokinetic parameters of rhein, herbal formulations containing different components may have a synergistic or antagonistic effect on rhein absorption. Considering its hepatotoxicity and nephrotoxicity, it is crucial to ensure that herbs rich in rhein are used in the appropriate formulations in TCM.

Researchers have begun to improve the absorption efficiency and bioavailability of rhein by altering the drug delivery route, using liposomes, nanoparticles, solid lipid nanoparticles, polymeric micelles, and hydrogels, among others (Cheng et al., 2021; Li et al., 2022). F127 modified liposomal rhein (F127-RPC-Lip) exhibits longer systemic circulation time, better drug distribution, and reduced pancreatic injury after complexation with lipids *via* non-covalent interactions. Wei et al. used an emulsification solvent evaporation technique to encapsulate rhein, which has poor water solubility, in mPEG-PLA NPs. RH-NPs significantly increase the plasma AUC of rhein, improve the biological half-life of rhein, increase the circulation time of rhein, and enhance renal permeability (Wei et al., 2017). Rhein (1.5%), Precrol ATO5 (2%), and lecinol (.5%) were prepared by hot homogenization followed by ultrasonication after co-solubilization in a mixture of organic solvent consisting of acetone/ethanol (1:1) to Rhein-SLNs. The AUC_0-t_ of rhein in the case of Rhein-SLNs was 2.06 times higher than that of the suspension group (Feng et al., 2017). DOX/RHE-loaded polymeric micelle, assembled by DSPE -PEG2000 and TPGS1000, released rhein in a rapid and targeted manner until 10 h to increase the distribution of rhein in tumor tissues (Han et al., 2018). Rhein hydrogel, directly self-assembled from rhein *via* intermolecular π–π interactions and hydrogen bonds, readily binds to Toll-like receptor four and then significantly dephosphorylates IκBα to inhibit the nuclear translocation of p65 in the NF-κB signaling pathway (Zheng et al., 2019). These new delivery systems improve the stability, absorption efficiency, and bioavailability of rhein.

## Concluding remarks

The effects of rhein on DM have been studied extensively. Rhein ameliorates IR by inhibiting the overactivity of HBP and the production of inflammatory cytokines through signaling pathways such as those of NF-κB and Wnt/β-catenin. Rhein inhibits ROS overproduction, thereby preventing oxidative stress in cells. It maintains the diversity of the gut microbiota to improve glucose metabolism. Moreover, it improves lipid metabolism, mitochondrial respiratory function, and pancreatic *ß*-cell function. The toxicology and pharmacokinetics of rhein have been studied in detail. In view of the low bioavailability and solubility of rhein, further structural modification studies can be conducted to search for derivatives with low toxicity and better efficacy that can be better applied in the treatment of DM.

## References

[B1] Ahmad F. B. Cisewski J. A. Minino A. Anderson R. N. (2021). Provisional mortality data — United States, 2020. MMWR Morb. Mortal. Wkly. Rep. 70 (14), 519–522. 10.15585/mmwr.mm7014e1 33830988 PMC8030985

[B2] American Diabetes Association (2018). Economic costs of diabetes in the U.S. In 2017. Diabetes Care 41 (5), 917–928. 10.2337/dci18-0007 29567642 PMC5911784

[B3] Bahiru E. Hsiao R. Phillipson D. Watson K. E. (2021). Mechanisms and treatment of dyslipidemia in diabetes. Curr. Cardiol. Rep. 23 (4), 26. 10.1007/s11886-021-01455-w 33655372

[B142] Barreto G. E. Sahebkar A. (2021). Pharmacological properties of plant-derived natural products and implications for human health (Advances in experimental medicine and biology, 1308). Springer. 10.1007/978-3-030-64872-5_17 33861448

[B4] Bati B. Celik I. Turan A. Eray N. Alkan E. E. Zirek A. K. (2020). Effect of isgin (Rheum ribes L.) on biochemical parameters, antioxidant activity and DNA damage in rats with obesity induced with high-calorie diet. Arch. Physiol. Biochem. 12, 1–9. 10.1080/13813455.2020.1819338 32924615

[B5] Bermudez B. Dahl T. B. Medina I. Groeneweg M. Holm S. Montserrat-de la Paz S. (2017). Leukocyte overexpression of intracellular NAMPT attenuates atherosclerosis by regulating pparγ-dependent monocyte differentiation and function. Arterioscler. Thromb. Vasc. Biol. 37 (6), 1157–1167. 10.1161/ATVBAHA.116.308187 28408371

[B6] Bironaite D. Ollinger K. (1997). The hepatotoxicity of rhein involves impairment of mitochondrial functions. Chem. Biol. Interact. 103 (1), 35–50. 10.1016/s0009-2797(96)03747-7 9051122

[B7] Cao C. Hui L. Li C. Yang Y. Zhang J. Liu T. (2019). *In vitro* study of the nephrotoxicity of total Dahuang (Radix Et Rhizoma Rhei Palmati) anthraquinones and emodin in monolayer human proximal tubular epithelial cells cultured in a transwell chamber. J. Tradit. Chin. Med. 39 (5), 609–623. 32186110

[B8] Cen B. Zhang T. Yuan J. Lu X. Xu J. Shi J. (2013). Protective effects of rhein on nonalcoholic fatty liver disease in rats fed with high fat diet. Chin. Archives Traditional Chin. Med. 31 (03), 545–547+709.

[B9] Chen W. Chang B. Zhang Y. Yang P. Liu L. (2015). Rhein promotes the expression of SIRT1 in kidney tissues of type 2 diabetic rat. Chin. J. Cell. Mol. Immunol. 31 (5), 615–619. 25940287

[B10] Cheng F. Cui H. Fang J. Yuan K. Guo Y. (2019). Ameliorative effect and mechanism of the purified anthraquinone-glycoside preparation from rheum palmatum L. On type 2 diabetes mellitus. Molecules 24 (8), 1454. 10.3390/molecules24081454 31013790 PMC6515271

[B11] Cheng L. Chen Q. Pi R. Chen J. (2021). A research update on the therapeutic potential of rhein and its derivatives. Eur. J. Pharmacol. 899, 173908. 10.1016/j.ejphar.2021.173908 33515540

[B12] Chi C. Jin M. Mu Y. Wang Ma X. Jia H. Yang L. (2008). Rhein improves insulin sensitivity of diabetic rats by increasing the protein expression levels of PPAR-γand GluT-4 in adipose tissue. Chin. J. Diabetes 16 (12), 746–748+758.

[B13] Chien S. C. Wu Y. C. Chen Z. W. Yang W. C. (2015). Naturally occurring anthraquinones: Chemistry and therapeutic potential in autoimmune diabetes. Evid. Based Complement. Altern. Med. 2015, 357357. 10.1155/2015/357357 PMC4381678 25866536

[B14] Chinese Pharmacopoeia Commission (2020). Pharmacopoeia of the People's Republic of China,Part II,2020. Beijing: China Medical Science and Technology Press.

[B15] Choi S. B. Ko B. S. Park S. K. Jang J. S. Park S. (2006). Insulin sensitizing and alpha-glucoamylase inhibitory action of sennosides, rheins and rhaponticin in Rhei Rhizoma. Life Sci. 78 (9), 934–942. 10.1016/j.lfs.2005.05.101 16182318

[B16] Chueakula N. Jaikumkao K. Arjinajarn P. Pongchaidecha A. Chatsudthipong V. Chattipakorn N. (2018). Diacerein alleviates kidney injury through attenuating inflammation and oxidative stress in obese insulin-resistant rats. Free Radic. Biol. Med. 115, 146–155. 10.1016/j.freeradbiomed.2017.11.021 29195834

[B17] Cooksey R. C. Hebert L. F. Jr Zhu J. H. Wofford P. Garvey W. T. McClain D. A. (1999). Mechanism of hexosamine-induced insulin resistance in transgenic mice overexpressing glutamine:fructose-6-phosphate amidotransferase: Decreased glucose transporter GLUT4 translocation and reversal by treatment with thiazolidinedione. Endocrinology 140 (3), 1151–1157. 10.1210/endo.140.3.6563 10067838

[B18] Dahms M. Lotz R. Lang W. Renner U. Bayer E. Spahn-Langguth H. (1997). Elucidation of phase I and phase II metabolic pathways of rhein: Species differences and their potential relevance. Drug Metab. Dispos. 25 (4), 442–452. 9107544

[B19] Danielsen K. Francis G. W. (1994). An alternative solvent system for the separation of anthraquinone aglycones from rhubarb on silica thin layers. Chromatographia 38 (7), 520. 10.1007/bf02269846

[B20] Deniyi A. Asase A. Ekpe P. K. Asitoakor B. K. Adu-Gyamfi A. Avekor P. Y. (2018). Ethnobotanical study of medicinal plants from Ghana;confirmation of ethnobotanical uses, and review of biological and toxicological studies on medicinal plants used in Apra Hills Sacred Grove. J. Herb. Med. 14, 76–87. 10.1016/j.hermed.2018.02.001

[B21] Ding Q. Ye C. Chen E. Zhang W. Wang X. (2018). Emodin ameliorates cartilage degradation in osteoarthritis by inhibiting NF-κB and Wnt/β-catenin signaling *in-vitro* and *in-vivo* . Int. Immunopharmacol. 61, 222–230. 10.1016/j.intimp.2018.05.026 29890416

[B22] Ding S. Xu S. Ma Y. Liu G. Jang H. Fang J. (2019). Modulatory mechanisms of the NLRP3 inflammasomes in diabetes. Biomolecules 9 (12), 850. 10.3390/biom9120850 31835423 PMC6995523

[B23] Dong L. Du H. Zhang M. Xu H. Pu X. Chen Q. (2022). Anti-inflammatory effect of Rhein on ulcerative colitis via inhibiting PI3K/Akt/mTOR signaling pathway and regulating gut microbiota. Phytother. Res. 36 (5), 2081–2094. 10.1002/ptr.7429 35229916

[B24] Dou F. Ding Y. Wang C. Duan J. Wang W. Xu H. (2020). Chrysophanol ameliorates renal interstitial fibrosis by inhibiting the TGF-β/Smad signaling pathway. Biochem. Pharmacol. 180, 114079. 10.1016/j.bcp.2020.114079 32511988

[B25] Du H. Shao J. Gu P. Lu B. Ye X. Liu Z. (2012). Improvement of glucose tolerance by rhein with restored early-phase insulin secretion in db/db mice. J. Endocrinol. Invest. 35 (6), 607–612. 10.1007/BF03345796 22776972

[B26] Duan S. Wu Y. Zhao C. Chen M. Yuan Y. Xing C. (2016). The wnt/β-catenin signaling pathway participates in rhein ameliorating kidney injury in DN mice. Mol. Cell Biochem. 411 (1-2), 73–82. 10.1007/s11010-015-2569-x 26346164

[B27] Duan S. F. Hu J. (2018). Effect of the Rhein Acid on the Expression of PPARγin diabetic rats′ mesangial cell. J. Pract. Med. 34 (10), 1636–1639.

[B28] Duan S. F. Hu J. Miu J. Du W. X. (2016). Effect of rheic acid on inflammatory cytokines in rats with diabetic nephropathy. Zhejiang J. Integr. Traditional Chin. West. Med. 26 (08), 714–716+781.

[B29] Fan Y. Niu Z. Xu C. Yang L. Yang T. (2019). Protic ionic liquids as efficient solvents in microwave-assisted extraction of rhein and emodin from rheum palmatum L. Rheum. palmatum L. Mol. 24 (15), 2770. 10.3390/molecules24152770 PMC6695579 31366111

[B30] Fang J. Y. Huang T. H. Chen W. J. Aljuffali I. A. Hsu C. Y. (2022). Rhubarb hydroxyanthraquinones act as antiobesity agents to inhibit adipogenesis and enhance lipolysis. Biomed. Pharmacother. 146, 112497. 10.1016/j.biopha.2021.112497 34891117

[B31] Feng H. Zhu Y. Fu Z. Li D. (2017). Preparation, characterization, and *in vivo* study of rhein solid lipid nanoparticles for oral delivery. Chem. Biol. Drug Des. 90 (5), 867–872. 10.1111/cbdd.13007 28432812

[B32] Gao Q. Qin W. S. Jia Z. H. Zheng J. M. Zeng C. H. Li L. S. (2010). Rhein improves renal lesion and ameliorates dyslipidemia in db/db mice with diabetic nephropathy. Planta Med. 76 (1), 27–33. 10.1055/s-0029-1185948 19639539

[B33] Gao Y. Chen X. Fang L. Liu F. Cai R. Peng C. (2014). Rhein exerts pro- and anti-inflammatory actions by targeting IKKβ inhibition in LPS-activated macrophages. Free Radic. Biol. Med. 72, 104–112. 10.1016/j.freeradbiomed.2014.04.001 24721152

[B34] García-Jiménez C. García-Martínez J. M. Chocarro-Calvo A. De la Vieja A. (2013). A new link between diabetes and cancer: Enhanced WNT/β-catenin signaling by high glucose. J. Mol. Endocrinol. 52 (1), R51–R66. 10.1530/JME-13-0152 24049067

[B35] Ge H. Tang H. Liang Y. Wu J. Yang Q. Zeng L. (2017). Rhein attenuates inflammation through inhibition of NF-κB and NALP3 inflammasome *in vivo* and *in vitro* . Drug Des. Devel Ther. 11, 1663–1671. 10.2147/DDDT.S133069 PMC5472410 28652704

[B36] Guo Q. (2021). Rhein improves exercise endurance of obese mice by up-regulating AMPK-Sirt1 signal pathway. Nanjing: Nanjing University.

[B37] Han N. N. Li X. Tao L. Zhou Q. (2018). Doxorubicin and rhein loaded nanomicelles attenuates multidrug resistance in human ovarian cancer. Biochem. Biophys. Res. Commun. 498 (1), 178–185. 10.1016/j.bbrc.2018.01.042 29317204

[B38] Hao K. Qi Q. Wan P. Zhang J. Hao H. Liang Y. (2014). Prediction of human pharmacokinetics from preclinical information of rhein, an antidiabetic nephropathy drug, using a physiologically based pharmacokinetic model. Basic Clin. Pharmacol. Toxicol. 114 (2), 160–167. 10.1111/bcpt.12148 24118734

[B39] Hashimoto T. Cook W. S. Qi C. Yeldandi A. V. Reddy J. K. Rao M. S. (2000). Defect in peroxisome proliferator-activated receptor alpha-inducible fatty acid oxidation determines the severity of hepatic steatosis in response to fasting. J. Biol. Chem. 275 (37), 28918–28928. 10.1074/jbc.M910350199 10844002

[B40] He A. Shen J. Xue Y. Xiang L. Li Y. Huang L. (2021). Diacerein attenuates vascular dysfunction by reducing inflammatory response and insulin resistance in type 2 diabetic rats. Biochem. Biophys. Res. Commun. 585, 68–74. 10.1016/j.bbrc.2021.11.017 34801936

[B41] He L. N. Yang A. H. Cui T. Y. Zhai Y. R. Zhang F. L. Chen J. X. (2015). Reactive metabolite activation by CYP2C19-mediated rhein hepatotoxicity. Xenobiotica 45 (4), 361–372. 10.3109/00498254.2014.984794 25815638

[B42] Hill M. A. Yang Y. Zhang L. Sun Z. Jia G. Parrish A. R. (2021). Insulin resistance, cardiovascular stiffening and cardiovascular disease. Metabolism 119, 154766. 10.1016/j.metabol.2021.154766 33766485

[B43] Hodgin J. B. Nair V. Zhang H. Randolph A. Harris R. C. Nelson R. G. (2013). Identification of cross-species shared transcriptional networks of diabetic nephropathy in human and mouse glomeruli. Diabetes 62 (1), 299–308. 10.2337/db11-1667 23139354 PMC3526018

[B44] Hou M. L. Chang L. W. Lin C. H. Lin L. C. Tsai T. H. (2014). Determination of bioactive components in Chinese herbal formulae and pharmacokinetics of rhein in rats by UPLC-MS/MS. Molecules 19 (4), 4058–4075. 10.3390/molecules19044058 24699148 PMC6271780

[B45] Hu G. Zhen Y. Luo G. Wei J. Lin Y. (2014). Rhein lysinate protects pancreas in type 2 diabetic mice. J. Army Med. Univ. 36 (05), 461–465.

[B46] Hu Y. (2012). Content determination of emodin and chrysophanol in sanhuangyou liniment by HPCE. Acta Chin. Med. 27 (08), 981–982.

[B47] Hu Y. Xiang L. Wang P. Lin B. Meng X. (2019). Hepatotoxicity and Nephrotoxicity of Rhei Radix et Rhizoma and Its Attenuation Methods. Chin. J. Exp. Traditional Med. Formulae 25 (11), 34–41.

[B48] Huang J. Chen W. L. Huang Y. F. Niu L. (2012). Effect of rhein on NADPH gene expression in the kidney of diabetic rats. Her. Med. 31 (10), 1285–1288.

[B49] Huang M. Ma J. Yang C. H. Lu B. Gu P. Shao J. Q. (2013). Effects of rheinic acid on markers of insulin secretion, inflammation and oxidative injury in db/db mice. Chin. Remedies Clin. 13 (08), 976–979+1109.

[B50] Huang Q. Lu G. Shen H. M. Chung M. C. Ong C. N. (2007). Anti-cancer properties of anthraquinones from rhubarb. Med. Res. Rev. 27 (5), 609–630. 10.1002/med.20094 17022020

[B51] Huang W. Xue A. Niu H. Jia Z. Wang J. (2009). Optimised ultrasonic-assisted extraction of flavonoids from Folium eucommiae and evaluation of antioxidant activity in multi-test systems *in vitro* . Food Chem. 114 (3), 1147–1154. 10.1016/j.foodchem.2008.10.079

[B52] Huang Y. F. Liu Z. H. Chen H. P. Zhou H. Wang J. P. Zhu M. Y. (2004). Improvement of diabetic metabolic disorders ameliorates renal lesions in db/db mice: Comparison between rhein and rosiglitazone[J]. Chin. J. Nephrol. Dialysis Transplant. 13, 215–221.

[B53] International Diabetes Federation (2021). IDF diabetes atlas. 10th ed. Brussels: International Diabetes Federation.

[B54] Jager S. Handschin C. St-Pierre J. Spiegelman B. M. (2007). AMP-activated protein kinase (AMPK) action in skeletal muscle via direct phosphorylation of PGC-1alpha. Proc. Natl. Acad. Sci. U. S. A. 104 (29), 12017–12022. 10.1073/pnas.0705070104 17609368 PMC1924552

[B55] Ji L. Gu H. (2021). The anti-obesity effects of rhein on improving insulin resistance (IR) and blood lipid levels are involved in endoplasmic reticulum stress (ERs), inflammation, and oxidative stress *in vivo* and vitro. Bioengineered 12 (1), 5797–5813. 10.1080/21655979.2021.1969196 34516329 PMC8806563

[B56] Jin M. (2008). Rhein improve the hyperglycemia,insulin sensitivity of diabetic rats and increases expressions of PPARγand GLUT-2 in hepatic tissue. Beijing: Chinese People's Liberation Army Medical College.

[B57] Khursheed R. Singh S. K. Wadhwa S. Kapoor B. Gulati M. Kumar R. (2019). Treatment strategies against diabetes: Success so far and challenges ahead. Eur. J. Pharmacol. 862, 172625. 10.1016/j.ejphar.2019.172625 31449807

[B58] Koyama J. Morita I. Kobayashi N. (2007). Simultaneous determination of anthraquinones in rhubarb by high-performance liquid chromatography and capillary electrophoresis. J. Chromatogr. A 1145 (1-2), 183–189. 10.1016/j.chroma.2007.01.076 17289060

[B59] Larsen N. Vogensen F. K. van den Berg F. W. Nielsen D. S. Andreasen A. S. Pedersen B. K. (2010). Gut microbiota in human adults with type 2 diabetes differs from non-diabetic adults. PLoS One 5 (2), e9085. 10.1371/journal.pone.0009085 20140211 PMC2816710

[B60] Lee J. H. Kim J. M. Kim C. (2003). Pharmacokinetic analysis of rhein in Rheum undulatum L. J. Ethnopharmacol. 84 (1), 5–9. 10.1016/s0378-8741(02)00222-2 12499069

[B61] Li J. Wang C. Han X. Liu S. Gao X. Guo C. (2022). Aramid nanofibers-reinforced rhein fibrous hydrogels as antibacterial and anti-inflammatory burn wound dressings. ACS Appl. Mater Interfaces 14 (40), 45167–45177. 10.1021/acsami.2c12869 36181475

[B62] Li K. J. Zhen Y. Z. (2017). Protective effects of lysine rhein on kidney of diabetic mice. J. North China Univ. Sci. Technol. Sci. Ed. 19 (03), 173–176.

[B63] Li Q. Su J. Jin S. J. Wei W. Cong X. D. Li X. X. (2018). Argirein alleviates vascular endothelial insulin resistance through suppressing the activation of Nox4-dependent O2- production in diabetic rats. Free Radic. Biol. Med. 121, 169–179. 10.1016/j.freeradbiomed.2018.04.573 29709706

[B64] Li Q. Verma I. M. (2002). NF-kappaB regulation in the immune system. Nat. Rev. Immunol. 2 (10), 725–734. 10.1038/nri910 12360211

[B65] Li Y. Liu Z. Liu D. Zhang J. Chen Z. Li L. (2001). Identification and function of glucose transporter 1 in human mesangial cells. Chin. Med. J. Engl. 114 (8), 824–828. 11780359

[B66] Liang J. Pei Y. Liu X. Qiu Q. Sun Y. Zhu Y. (2015). The CDKAL1 gene is associated with impaired insulin secretion and glucose-related traits: The cardiometabolic risk in Chinese (CRC) study. Clin. Endocrinol. (Oxf). 83 (5), 651–655. 10.1111/cen.12838 26119585

[B68] Lin C. M. (2004). Beta-catenin controls hair follicle morphogenesis and stem cell differentiation. J. Med. Postgraduates (04), 358–360.

[B69] Lin M. L. Chen S. S. Lu Y. C. Liang R. Y. Ho Y. T. Yang C. Y. (2007). Rhein induces apoptosis through induction of endoplasmic reticulum stress and Ca2+-dependent mitochondrial death pathway in human nasopharyngeal carcinoma cells. Anticancer Res. 27 (5A), 3313–3322. 17970076

[B70] Lin Y. J. Hu G. Li K. J. Zhao Y. F. Wei J. Zhen Y. Z. (2015). The protection of Rhein lysinate to liver in diabetic mice induced by high-fat diet and streptozotocin. Arch. Pharm. Res. 38 (5), 885–892. 10.1007/s12272-014-0423-4 24968924

[B71] Lin Y. J. Zhen Y. Z. Wei J. B. Wei J. Dai J. Gao J. L. (2017). Rhein lysinate protects renal function in diabetic nephropathy of KK/HlJ mice. Exp. Ther. Med. 14 (6), 5801–5808. 10.3892/etm.2017.5283 29285124 PMC5740561

[B72] Liu J. Chen Z. Zhang Y. Zhang M. Zhu X. Fan Y. (2013). Rhein protects pancreatic β-cells from dynamin-related protein-1-mediated mitochondrial fission and cell apoptosis under hyperglycemia. Diabetes 62 (11), 3927–3935. 10.2337/db13-0251 23919963 PMC3806614

[B73] Liu Q. (2009). The establishment of hGFAT inhibitor Screening method & the experimental studies of Rhein on improving insulin resistance and lipid metabolic disorder. Beijing: Chinese Academy of Medical Sciences & Peking Union Medical College.

[B74] Liu Q. Yu S. Zhu Y. Gao T. (2011). Effects of rhein treatment on resistin mRNA expression of adipose tissue and plasma free fatty acid in diabetic rats. Chin. J. Diabetes 19 (05), 347–349.

[B75] Liu Z. H. Li Y. J. Chen Z. H. Liu D. Li L. S. (2001a). Glucose transporter in human glomerular mesangial cells modulated by transforming growth factor-beta and rhein. Acta Pharmacol. Sin. 22 (2), 169–175. 11741523

[B76] Liu Z. H. Li Y. J. Zhu J. M. Liu D. Guo D. Chen Z. H. (2001b). The effect of glucose transporter 1 on hexosamine biosynthesis pathway in rat glomerular mesangial cells. Chin. J. Endocrinol. Metabolism (06), 46–49.

[B77] Liu Z. H. Liu D. Zhu J. M. Guo X. H. Li L. S. (2000). High glucose-induced mesangial cells transforming growth factorβ1 synthesis is mediated by upregulated hexosamine biosynthesis pathway. Chin. J. Nephrol. Dialysis Transplant. (04), 303–310.

[B78] Lupi R. Del Prato S. (2008). Beta-cell apoptosis in type 2 diabetes: Quantitative and functional consequences. Diabetes Metab. 34 (2), S56–S64. 10.1016/S1262-3636(08)73396-2 18640587

[B79] Ma X. Chen Y. Hui R. (1989). Analysis of anthraquinones in Rheum franzenbachii Münt (rhubarb) by thin-layer chromatography. Chromatographia 27 (9), 465–466. 10.1007/bf02319565

[B80] Malaguti C. Vilella C. A. Vieira K. P. Souza G. H. Hyslop S. Zollner Rde L. (2008). Diacerhein downregulate proinflammatory cytokines expression and decrease the autoimmune diabetes frequency in nonobese diabetic (NOD) mice. Int. Immunopharmacol. 8 (6), 782–791. 10.1016/j.intimp.2008.01.020 18442781

[B81] Mao Y. Zhang M. Yang J. Sun H. Wang D. Zhang X. (2017). The UCP2-related mitochondrial pathway participates in rhein-induced apoptosis in HK-2 cells. Toxicol. Res. (Camb) 6 (3), 297–304. 10.1039/c6tx00410e 30090499 PMC6062232

[B82] Marshall S. Bacote V. Traxinger R. R. (1991). Discovery of a metabolic pathway mediating glucose-induced desensitization of the glucose transport system. Role of hexosamine biosynthesis in the induction of insulin resistance. J. Biol. Chem. 266 (8), 4706–4712. 10.1016/s0021-9258(19)67706-9 2002019

[B83] Masson E. Lagarde M. Wiernsperger N. El Bawab S. (2006). Hyperglycemia and glucosamine-induced mesangial cell cycle arrest and hypertrophy: Common or independent mechanisms? IUBMB Life 58, 381–388. 10.1080/15216540600755980 16801212

[B84] Masson E. Wiernsperger N. Lagarde M. El Bawab S. (2005). Glucosamine induces cell-cycle arrest and hypertrophy of mesangial cells: Implication of gangliosides. Biochem. J. 388 (2), 537–544. 10.1042/BJ20041506 15654767 PMC1138961

[B85] Navarro J. F. Mora C. (2005). Role of inflammation in diabetic complications. Nephrol. Dial. Transpl. 20 (12), 2601–2604. 10.1093/ndt/gfi155 16188894

[B86] Neuschwander-Tetri B. A. Caldwell S. H. (2003). Nonalcoholic steatohepatitis: Summary of an AASLD single topic conference. Hepatology 37 (5), 1202–1219. 10.1053/jhep.2003.50193 12717402

[B87] Nishikawa T. Kukidome D. Sonoda K. Fujisawa K. Matsuhisa T. Motoshima H. (2007). Impact of mitochondrial ROS production in the pathogenesis of insulin resistance. Diabetes Res. Clin. Pract. 77 (3), S161–S164. 10.1016/j.diabres.2007.01.071 17481767

[B88] Park E. Y. Kim H. J. Kim Y. K. Park S. U. Choi J. E. Cha J. Y. (2012). Increase in insulin secretion induced by panax ginseng berry extracts contributes to the amelioration of hyperglycemia in streptozotocininduced diabetic mice. J. Ginseng Res. 36 (2), 153–160. 10.5142/jgr.2012.36.2.153 23717115 PMC3659577

[B89] Park K. Lee K. Zhang B. Zhou T. He X. Gao G. (2011). Identification of a novel inhibitor of the canonical Wnt pathway. Mol. Cell Biol. 31 (14), 3038–3051. 10.1128/MCB.01211-10 21576363 PMC3133395

[B90] Peigen X. Liyi H. Liwei W. (1984). Ethnopharmacologic study of Chinese rhubarb. J. Ethnopharmacol. 10 (3), 275–293. 10.1016/0378-8741(84)90016-3 6748707

[B91] Pharmacopoeia of the People’s Republic of China (2005). First div. Beijing: China Chemical Industry Press, 98–145.

[B92] Piovesan F. Tres G. S. Moreira L. B. Andrades M. E. Lisboa H. K. Fuchs S. C. (2017). Effect of diacerein on renal function and inflammatory cytokines in participants with type 2 diabetes mellitus and chronic kidney disease: A randomized controlled trial. PLoS One 12 (10), e0186554. 10.1371/journal.pone.0186554 29049415 PMC5648185

[B93] Pires B. R. B. Silva R. C. M. C. Ferreira G. M. Abdelhay E. (2018). NF-kappaB: Two sides of the same coin. Genes (Basel) 9 (1), 24. 10.3390/genes9010024 29315242 PMC5793177

[B94] Pitocco D. Zaccardi F. Di Stasio E. Romitelli F. Santini S. A. Zuppi C. (2010). Oxidative stress, nitric oxide, and diabetes. Rev. Diabet. Stud. 7 (1), 15–25. 10.1900/RDS.2010.7.15 20703435 PMC2923377

[B95] Qi L. W. Liu E. H. Chu C. Peng Y. B. Cai H. X. Li P. (2010). Anti-diabetic agents from natural products--an update from 2004 to 2009. Curr. Top. Med. Chem. 10 (4), 434–457. 10.2174/156802610790980620 20180758

[B96] Qin J. Li Y. Cai Z. Li S. Zhu J. Zhang F. (2012). A metagenome-wide association study of gut microbiota in type 2 diabetes. Nature 490 (7418), 55–60. 23023125 10.1038/nature11450

[B97] Rhein P. Desjardins E. M. Rong P. Ahwazi D. Bonhoure N. Stolte J. (2021). Compound- and fiber type-selective requirement of AMPKγ3 for insulin-independent glucose uptake in skeletal muscle. Mol. Metab. 51, 101228. 10.1016/j.molmet.2021.101228 33798773 PMC8381060

[B98] Rhubarb (2006). Drugs and lactation database (LactMed) [internet]. Bethesda (MD): National Library of Medicine US.

[B99] Scott J. A. King G. L. (2004). Oxidative stress and antioxidant treatment in diabetes. Ann. N. Y. Acad. Sci. 1031, 204–213. 15753146 10.1196/annals.1331.020

[B100] Shen C. Zhang Z. Xie T. Ji J. Xu J. Lin L. (2020). Rhein suppresses lung inflammatory injury induced by human respiratory syncytial virus through inhibiting NLRP3 inflammasome activation via NF-κB pathway in mice. Front. Pharmacol. 28 (10), 1600. 10.3389/fphar.2019.01600 PMC6997271 32047436

[B101] Sheng X. Wang M. Lu M. Xi B. Sheng H. Zang Y. Q. (2011). Rhein ameliorates fatty liver disease through negative energy balance, hepatic lipogenic regulation, and immunomodulation in diet-induced obese mice. Am. J. Physiol. Endocrinol. Metab. 300 (5), E886–E893. 10.1152/ajpendo.00332.2010 21364120

[B102] Shoelson S. E. Lee J. Goldfine A. B. (2006). Inflammation and insulin resistance. J. Clin. Invest. 116 (7), 1793–1801. 10.1172/JCI29069 16823477 PMC1483173

[B103] Sun C. Liu H. (2008). Application of non-ionic surfactant in the microwave-assisted extraction of alkaloids from Rhizoma coptidis. Anal. Chim. Acta 612 (2), 160–164. 10.1016/j.aca.2008.02.040 18358861

[B104] Sun H. Yang J. Mao Y. Wang D. Yu F. (2015). Involvement of Fas-dependent pathway in rhein-induced apoptosis of HK-2 cells. Nanjing: Journal of China Pharmaceutical University, 469–475.

[B105] Takizawa Y. Morota T. Takeda S. Aburada M. (2003). Pharmacokinetics of rhein from Onpi-to, an Oriental herbal medicine, in rats. Biol. Pharm. Bull. 26 (5), 613–617. 10.1248/bpb.26.613 12736499

[B106] Tobar N. Oliveira A. G. Guadagnini D. Bagarolli R. A. Rocha G. Z. Araújo T. G. (2011). Diacerhein improves glucose tolerance and insulin sensitivity in mice on a high-fat diet. Endocrinology 152 (11), 4080–4093. 10.1210/en.2011-0249 21896669

[B107] Tres G. S. Fuchs S. C. Piovesan F. Koehler-Santos P. Pereira F. D. S. Camey S. (2018). Effect of diacerein on metabolic control and inflammatory markers in patients with type 2 diabetes using antidiabetic agents: A randomized controlled trial. J. Diabetes Res. 2018, 4246521. 10.1155/2018/4246521 29805981 PMC5902058

[B108] Tu Y. Gu L. Chen D. Wu W. Liu H. Hu H. (2017). Rhein inhibits autophagy in rat renal tubular cells by regulation of AMPK/mTOR signaling. Sci. Rep. 7, 43790. 10.1038/srep43790 28252052 PMC5333140

[B109] Wajchenberg B. L. (2007). beta-cell failure in diabetes and preservation by clinical treatment. Endocr. Rev. 28 (2), 187–218. 10.1210/10.1210/er.2006-0038 17353295

[B110] Wang J. B. Kong W. J. Wang H. J. Zhao H. P. Xiao H. Y. Dai C. M. (2011). Toxic effects caused by rhubarb (Rheum palmatum L.) are reversed on immature and aged rats. J. Ethnopharmacol. 134 (2), 216–220. 10.1016/j.jep.2010.12.008 21163343

[B111] Wang M. Yu S. Liu Q. (2011). Effect of rhein on oxidative stress of the kidneys in fat diabetic rats. Chin. Archives Traditional Chin. Med. 29 (07), 1559–1560.

[B112] Wang R. Lei H. Zang P. Du H. (2016). The effect of rhein on the gut microbiota in diabetes mice. Chin. J. Microecology 28 (01), 21–24+46.

[B113] Wang R. (2016). Study about the intestinal mechanism involved in the anti-hyperglycemic effect of rhein. Nanjing: Nanjing University.

[B114] Wang R. Zang P. Chen J. Wu F. Zheng Z. Ma J. (2018). Gut microbiota play an essential role in the antidiabetic effects of rhein. Evid. Based Complement. Altern. Med. 2018, 6093282. 10.1155/2018/6093282 PMC6077525 30108658

[B115] Wei J. Zhen Y. Z. Cui J. He F. Shen T. Hu G. (2016). Rhein lysinate decreases inflammation and adipose infiltration in KK/HlJ diabetic mice with non-alcoholic fatty liver disease. Arch. Pharm. Res. 39 (7), 960–969. 10.1007/s12272-016-0770-4 27277164

[B116] Wei Y. Luo X. Guan J. Ma J. Zhong Y. Luo J. (2017). Biodegradable nanoparticles for improved kidney bioavailability of rhein: Preparation, characterization, plasma, and kidney pharmacokinetics. Drug Dev. Ind. Pharm. 43 (11), 1885–1891. 10.1080/03639045.2017.1353519 28692315

[B117] Wu X. Duan W. Xia P. Wang Y. Bian H. Zhao L. (2022). Rapid screening of active components from Rhei Radix et Rhizoma for hypolipidemia by L02 hepatic steatosis cell membrane. Chin. Traditional Herb. Drugs 53 (03), 735–742.

[B118] Xie H. C. Shang J. (2014). Study on the extraction process of total anthraquinones in Radix et Rhizoma Rhei and their antilipemic effects. Afr. J. Tradit. Complement. Altern. Med. 11 (2), 358–362. 10.4314/ajtcam.v11i2.22 25435622 PMC4202646

[B119] Yang D. Huang W. Y. Li Y. Q. Chen S. Y. Su S. Y. Gao Y. (2022). Acute and subchronic toxicity studies of rhein in immature and d-galactose-induced aged mice and its potential hepatotoxicity mechanisms. Drug Chem. Toxicol. 45 (3), 1119–1130. 10.1080/01480545.2020.1809670 32842782

[B120] Yang D. (2022). Study on MRP-oxidative stress-based hepatotoxicity and related mechanisms of rhein. Chengdu: Chengdu University of Traditional Chinese Medicine.

[B121] Yang L. Liu T. Huang Z. Li J. Wu L. (2011). Research progress on the mechanism of single-Chinese medicinal herbs in treating diabetes mellitus. Chin. J. Integr. Med. 17, 235–240. 10.1007/s11655-010-0674-6 21359928

[B122] Yang X. Geng H. You L. Yuan L. Meng J. Ma Y. (2022). Rhein protects against severe acute pancreatitis *in vitro* and *in vivo* by regulating the JAK2/STAT3 pathway. Front. Pharmacol. 13, 778221. 10.3389/fphar.2022.778221 35370748 PMC8969574

[B123] Ye M. Han J. Chen H. Zheng J. Guo D. (2007). Analysis of phenolic compounds in rhubarbs using liquid chromatography coupled with electrospray ionization mass spectrometry. J. Am. Soc. Mass Spectrom. 18 (1), 82–91. 10.1016/j.jasms.2006.08.009 17029978

[B124] You L. Dong X. Yin X. Yang C. Leng X. Wang W. (2018). Rhein induces cell death in HepaRG cells through cell cycle arrest and apoptotic pathway. Int. J. Mol. Sci. 19 (4), 1060. 10.3390/ijms19041060 29614833 PMC5979559

[B125] Zeng C. Liu X. Chen G. Wu Q. Liu W. Luo H. (2014). The molecular mechanism of rhein in diabetic nephropathy. Evid. Based Complement. Altern. Med. 2014, 487097. 10.1155/2014/487097 PMC4243766 25435889

[B126] Zeng L. N. Ma Z. J. Zhao Y. L. Zhang L. D. Li R. S. Wang J. B. (2013). The protective and toxic effects of rhubarb tannins and anthraquinones in treating hexavalent chromium-injured rats: The yin/yang actions of rhubarb. J. Hazard Mater 246-247, 1–9. 10.1016/j.jhazmat.2012.12.004 23276788

[B127] Zhang D. Yang X. Li J. Yu J. Wu X. (2019). Effect of hyperinsulinaemia and insulin resistance on endocrine, metabolic and fertility outcomes in women with polycystic ovary syndrome undergoing ovulation induction. Clin. Endocrinol. (Oxf). 91 (3), 440–448. 10.1111/cen.14050 31222771

[B129] Zhang T. Zhu X. Cheng H. Zhang M. Lv S. (2015). Effects of rhein on expressions of Wnt/β-catenin protein and TGF-β1 in high glucose induced human mesangial cell. Glob. Tradit. Chin. Med. 8 (S2), 155–156.

[B130] Zhang Y. X. Li J. S. Peng W. W. Liu X. Yang G. M. Chen L. H. (2013). Comparative pharmacokinetics of aloe-emodin, rhein and emodin determined by liquid chromatography-mass spectrometry after oral administration of a rhubarb peony decoction and rhubarb extract to rats. Pharmazie 68 (5), 333–339. 23802430

[B131] Zhao D. Feng S. X. Zhang H. J. Zhang N. Liu X. F. Wan Y. (2021). Pharmacokinetics, tissue distribution and excretion of five rhubarb anthraquinones in rats after oral administration of effective fraction of anthraquinones from rheum officinale. Xenobiotica 51 (8), 916–925. 10.1080/00498254.2021.1940353 34110981

[B132] Zhao W. Zhang X. Zhang R. Zhang K. Li Y. Xu F. J. (2020). Self-assembled herbal medicine encapsulated by an oxidation-sensitive supramolecular hydrogel for chronic wound treatment. ACS Appl. Mater Interfaces 12 (51), 56898–56907. 10.1021/acsami.0c19492 33296174

[B133] Zheng J. Fan R. Wu H. Yao H. Yan Y. Liu J. (2019). Directed self-assembly of herbal small molecules into sustained release hydrogels for treating neural inflammation. Nat. Commun. 10 (1), 1604. 10.1038/s41467-019-09601-3 30962431 PMC6453967

[B134] Zheng J. Zhu J. Li L. Liu Z. (2008). Rhein reverses the diabetic phenotype of mesangial cells over-expressing the glucose transporter (GLUT1) by inhibiting the hexosamine pathway. Br. J. Pharmacol. 153 (7), 1456–1464. 10.1038/bjp.2008.26 18264122 PMC2437903

[B135] Zheng Z. (2020). Analysis of the hypoglycemic effect of rhein based on the structural characteristics of gut microbiota in type 2 diabetes mellitus. Guangzhou: Southern Medical University.

[B136] Zhou H. Song F. R. Liu Z. Q. Zheng Y. N. Liu S. Y. (2009). Studies on the components of unprocessed and processed radixet rhizoma rhei, cortex phellodendri and radix paeoniae rubraby HPLC-UV and ESI-MS. Chin. J. Pharm. Analysis 29 (06), 883–888.

[B137] Zhou Y. Gao C. Vong C. Tao H. Li H. Wang S. (2022). Rhein regulates redox-mediated activation of NLRP3 inflammasomes in intestinal inflammation through macrophage-activated crosstalk. Br. J. Pharmacol. 179 (9), 1978–1997. 10.1111/bph.15773 34882785

[B138] Zhu J. Liu Z. Huang H. Chen Z. Li L. (2003). Rhein inhibits transforming growth factor beta1 induced plasminogen activator inhibitor-1 in endothelial cells. Chin. Med. J. Engl. 116 (3), 354–359. 12781036

[B139] Zhu J. Liu Z. Li Y. Liu D. Guo X. Li L. (2001). Inhibition of glucose transporter 1 overexpression in mesangial cells by rhein. Chin. J. Intern. Med. 08, 36–41+77. 11718055

[B140] Zhu J. Xu D. Yang R. Liu M. Liu Y. (2020). The triglyceride glucose index (TyG) and CDKAL1 gene rs10946398 SNP are associated with NAFLD in Chinese adults. Minerva Endocrinol. 10.23736/S0391-1977.20.03273-3 33269568

[B141] Zhuang S. Zhong J. Bian Y. Fan Y. Chen Q. Liu P. (2019). Rhein ameliorates lipopolysaccharide-induced intestinal barrier injury via modulation of Nrf2 and MAPKs. Life Sci. 216, 168–175. 10.1016/j.lfs.2018.11.048 30471284

